# Content and Availability of Minerals in Plant-Based Burgers Compared with a Meat Burger

**DOI:** 10.3390/nu15122732

**Published:** 2023-06-13

**Authors:** Gladys O. Latunde-Dada, Naroa Kajarabille, Sophie Rose, Sarah M. Arafsha, Tugba Kose, Mohamad F. Aslam, Wendy L. Hall, Paul A. Sharp

**Affiliations:** 1 Department of Nutritional Sciences, School of Life Course Sciences, King’s College London, Franklin-Wilkins-Building, 150 Stamford Street, London SE1 9NH, UK; naroa.kajarabille@ehu.eus (N.K.); sophie.rose@kcl.ac.uk (S.R.); sarah.arafsha@kcl.ac.uk (S.M.A.); tugba.kose@kcl.ac.uk (T.K.); mf.aslam@kcl.ac.uk (M.F.A.); wendy.hall@kcl.ac.uk (W.L.H.); paul.a.sharp@kcl.ac.uk (P.A.S.); 2 Department of Preventive Medicine and Public Health, University of the Basque Country (UPV/EHU), 01006 Vitoria, Spain; 3 Nutrition and Obesity Group, Department of Pharmacy and Food Science, Lucio Lascaray Research Institute, University of the Basque Country (UPV/EHU), 01006 Vitoria, Spain

**Keywords:** iron, burgers, plant, bioaccessibility, bioavailability

## Abstract

Increasing numbers of individuals follow plant-based diets. This has sparked interest in the nutritional evaluation of the meat substitute sector. Nutritional understanding of these products is vital as plant-based eating becomes more common. For example, animal products are rich sources of iron and zinc, and plant-based foods could be inadequate in these minerals. The main aim was to analyse the mineral composition and absorption from a range of plant-based meat-free burgers and compare them to a typical beef burger. Total and bioaccessible mineral contents of plant-based burgers and a beef burger were determined using microwave digestion and in vitro simulated gastrointestinal digestion, respectively. Mineral bioavailability was analysed by in vitro simulated gastrointestinal digestion of foods, followed by exposure of Caco-2 cells to the sample digests and assessment of mineral uptake. Mineral quantification for all samples was achieved using inductively coupled ICP-optical emission spectrometry (ICP-OES). The content of minerals varied significantly amongst the burgers. Significantly greater quantities of Fe and Zn were found in the beef burger compared to most meat substitutes. Bioaccessible Fe was significantly higher in the beef compared to most of the plant-based meat alternatives; however, bioavailable Fe of most plant-based burgers was comparable to beef (*p* > 0.05). Similarly, bioaccessible Zn was significantly (*p* < 0.001) higher from the beef burger. Moreover, beef was superior regarding bioavailable Zn (*p* ≤ 0.05–0.0001), with only the mycoprotein burger displaying comparable Zn bioavailability (*p* > 0.05). Beef is an excellent source of bioaccessible Fe and Zn compared to most plant-based substitutes; however, these plant-based substitutes were superior sources of Ca, Cu, Mg and Mn. The quantity of bioaccessible and absorbable Fe varies dramatically among the meat alternatives. Plant-based burgers have the potential to provide adequate quantities of iron and zinc to those consuming such burgers as part of a varied diet. Thus, guiding consumer choices will depend on the variety of the vegetable constituents and their iron nutritional quality in different burgers.

## 1. Introduction

To meet the United Nations Sustainable Development Goals and Paris Agreement, it may be necessary for global diets to shift to embrace greater reliance on plant-based foods. According to the EAT-Lancet Commission, to help meet global targets by 2050, a dietary pattern that is healthy for both people and planet should double the consumption of fruits, vegetables, legumes and nuts, and reduce by 50% the consumption of added sugars and red meat [1]. These practices could support environmental sustainability, alongside potential health benefits for the population. For instance, those who choose to follow a vegetarian or vegan diet are reported to have a lower risk of cardiovascular disease, type 2 diabetes, and obesity [2]. In general, though iron stores of vegetarians may be low, anaemia incidences may not differ significantly from those of omnivores [3]. Hence, a well-planned varied vegetarian diet could maintain optimal iron nutrition in the populace. In addition, a plant-based diet appears to be beneficial for human health by promoting the development of more diverse and stable microbial gut populations [4].

Although vegetarian and vegan diets have been claimed to have a variety of positive health effects, there is much debate about whether they can match an omnivorous diet in terms of nutrient intake [5]. Minerals of concern are calcium, iron, and zinc, as well as vitamin B12, vitamin D, and omega-3 fatty acids [6]. As these micronutrients are found abundantly in animal-derived products, vegetarian and vegan diets must be properly guided to avoid nutritional deficiencies.

Iron deficiency is the most common mineral insufficiency worldwide and is estimated to affect two billion people globally [7,8]. The importance of iron cannot be understated, as iron inadequacy can have devastating and irreversible consequences, such as impaired cognitive development during childhood [9]. Those on a vegetarian or vegan diet, are at a risk of iron deficiency if they do not follow professional dietary guidelines, because the iron obtained is predominantly in the form of nonheme iron [10]. On the other hand, an omnivorous diet contains a combination of heme (from meat and fish products) as well as nonheme iron (from plant products). Heme iron is absorbed more efficiently from a human diet (20–30%) than nonheme iron (5–15%); thus, heme iron demonstrates greater bioavailability [11].

The bioavailability of nonheme iron varies in different foods, and this is largely influenced by endogenous constituent inhibitors and promotors within these foods. The main inhibitor of iron absorption is phytic acid, whilst polyphenols, tannins, calcium, and soy also play inhibitory roles [12,13]. In contrast, ascorbic acid, meat, and citric acid are all enhancers of iron absorption [14]. Ascorbic acid in the diet reduces ferric iron into ferrous iron, which has a greater capacity for absorption [15]. In addition to iron, a large proportion of zinc is derived from animal products, with meat constituting a third of the zinc intake in UK diets (National Diet and Nutrition Survey NDNS) [16]. As meat is eliminated from the diet, it is replaced by an increase in phytate-rich foods such as legumes and wholegrains, which can have negative effects on the ability to absorb both minerals. Thus, while those following vegetarian or vegan diets can acquire minerals from plant products, their absorption is largely inhibited by phytic acid [17].

The change in consumer needs, i.e., the greater requirement for vegetarian and vegan food products, has not gone unnoticed by retailers in the UK. The vegetarian and vegan market has experienced substantial growth, particularly in the ready meals and convenience food sectors [5]. It is estimated that meat analogues contribute significantly to human diets which exclude animal flesh and their derivatives [18]. Meat-alternative products are aimed primarily at vegetarians, vegans, pescetarians, or flexitarians, as well as those seeking to diversify their diets [19]. Meat substitutes are largely based on soya, mycoprotein, vegetables, wheat derivatives, and pulses [20,21]. These meat analogues often mimic products traditionally prepared from animals, as they can be manufactured into burger, meatball, and sausage formats.

Plant-based meat substitutes as ready meal products are also widely consumed by adults due to their convenience as controlled portions (single servings help consumers manage their meal intake) and easy cooking by microwave, and they offer a variety of choices of food types. These products have been reported to be nutritionally adequate, as well as having some shortfalls [22,23,24]. For example, a fava bean protein substitute for beef or fish exhibited reduced iron bioavailability in healthy females [25].

There are limited data assessing whether plant-based meat alternatives can match the high mineral profile of comparable nonvegetarian meals, and this could have important implications on the mineral statuses of individuals who do not consume meat products. Arising from this, the main purpose of this study was to assess whether plant-based burgers can compare with the mineral content and bioavailability of their meat-based equivalents to inform consumers about their choices. Consequently, the study determined the mineral content and bioaccessibility and bioavailability of iron and zinc as a novel approach, in both vegetarian and vegan burgers, and compared them to those of a beef burger.

## 2. Materials and Methods

### 2.1. Reagents and Chemicals

Unless otherwise stated, all the reagents and chemicals used in this study were purchased from Sigma-Aldrich Ltd. (Dorset, UK). Solutions of enzymes (pepsin and pancratic-bile extract) were all freshly prepared just before use.

### 2.2. Sample Collection and Preparation

Burger samples were sourced from a major supermarket in the UK. Samples were cooked as per the manufacturers’ instructions using an oven or a grill. Each burger (50 g) was placed in an aluminium dish, frozen at −20 °C, and freeze-dried for five days using a freeze dryer (CHRIST ALPHA 1-4 L Dplus SciQuip Ltd, Newtown, UK). Dried samples were ground in a blender to obtain a fine powder, stored in sealed bags, and kept at 5 °C.

### 2.3. Moisture Content Analysis

To determine the dry matter, 5 g of each burger was placed in a crucible and dried in an oven at 65 °C for 48 h until constant weight was attained and transferred to desiccators. Dried samples were reweighed, and moisture content was calculated. The data are presented as means *n* = 5 ± SEM.

### 2.4. Total Mineral Content of Samples

The total mineral content of each sample was determined by inductively coupled plasma-optical emission spectroscopy (ICP-OES) (MS) after microwave digestion as described [26]. Essentially, 0.5 g of each sample was placed into digestion to which 10 mL of 70% 1 M HNO_3_ was added. Tubes were loaded into a MARS 6 microwave digestion system (CEM Microwave Technology Ltd., Buckingham, UK) and the temperature was raised to 210 °C for 45 min, and then cooled to room temperature. For ICP-OES, samples were decanted into tubes and 140 μL of 1 ppm yttrium was added; thereafter, the volume was made up to 14 mL using Milli-Q water. For ICP-MS, 50 μL of each sample was added to a solution consisting of 4.9 mL Milli-Q water and 50 μL of 5 ppm gallium. The data are presented as means *n* = 5 ± SEM.

### 2.5. In Vitro Simulated Gastrointestinal Digestion of Samples

An in vitro simulated enzymatic digestion of samples was employed to mimic gastrointestinal conditions and assess bioaccessibility of minerals [27,28,29]. In brief, 0.5 g of sample was mixed with 10 mL of saline solution (140 mM NaCl; 5 mM KCl, pH 2.0) and left for 15 min in the dark (20 °C). The pH was adjusted (HANNA Instrument) to 2.0 using 1 M HCl, and 0.5 mL of pepsin (16 mg/mL) from porcine gastric mucosa was added. Samples were incubated for 90 min in the dark at 37 °C on a rocking platform (Stuart Scientific Shaker) set to 150 rpm. The pH was then altered to 7.0 using 1 M NaHCO_3_ before adding 2.5 mL porcine bile and pancreatin extracts (8.5 mg/mL bile; 1.4 mg/mL pancreatin) was added. Volumes were raised to 15 mL using pH 7.0 saline solution and then incubated on the rocking platform as described earlier. Thereafter, samples were centrifuged (Eppendorf Centrifuge 5804R, Sigma) at 5000 rpm for 10 min. Supernatants were decanted and filtered overnight in the dark at 5 °C using Whatman 540 filter paper, and filtrates were stored at −20 °C until used. The procedure was also performed for sham controls to which no food sample was added. The data are presented as means *n* = 3 ± SEM.

### 2.6. Mineral Uptake by Caco-2 Cells

Caco-2 cells were seeded into six-well plates and grown for 14 days under the conditions to allow for full differentiation. On the day before experimentation, the culture medium was discarded and replaced with 2 mL/well of serum-free RPMI-1640 medium. The next day, digests were defrosted, heated to 100 °C for 5 min to inactivate enzymes, and then centrifuged for 5 min at 5000 rpm (Eppendorf Centrifuge 5804R, Sigma). Cell medium was removed and replaced with 2 mL/well of working solution, which consisted of 1 part digest to 1 part serum-free medium. Cells were incubated for 1 h for ICP-OES analysis and 4 h for ICP-MS analysis (5% CO_2_; 37 °C).

For ICP-OES analysis, working solutions were removed, wells were washed twice with Versene, and 500 μL/well of 50 mM NaOH was added. Plates were placed on a rocking platform at 20 °C for 2 h. For lysis, cells were subjected to 45 min of sonication using a heated (40 °C) ultrasonic bath (FB 11021, Fisherbrand). To adjust for variability in cell number between samples, 20 μL aliquots of each lysate were transferred to 96-well plates for protein quantification. The remaining lysates were desiccated for 2 h at 60 °C using a vacuum-integrated centrifugal concentrator to minimise variations due to volume differences (miVAC DNA-23050-A00, Genevac). Dried lysates were mixed with 250 μL of 65% HNO_3_, and then 200 μL aliquots were made up to 6 mL using 17% HNO_3_ (which contained 1.0175 mL of 1000 ppm yttrium per 1 L of acid). Samples were digested at 80 °C for 12 h and left to cool before analysis by ICP-OES. The data are presented as means *n* = 4 ± SEM.

### 2.7. Protein Analysis

A Bio-Rad protein assay kit (Bio-Rad Laboratories, UK) was utilised to determine the protein from Caco-2 cell lysate. To a 96-well microtiter plate, 20 μL of the sample, 20 μL of reagent A + S (1 mL of solution A added to 20 mL), and 160 μL of reagent B were added. Bovine serum albumin was used as a standard. The plate was incubated at room temperature on a platform shaker for 10 min and absorbance was read at 750 nm. A standard curve was plotted, and protein content was extrapolated and calculated. 

### 2.8. Statistical Analysis

Data were analysed using Microsoft Office Excel 2017 and GraphPad Prism 8.0. Data are expressed as mean ± standard error of the mean (SEM). Comparisons of means were analysed by one-way analysis of variance (ANOVA). Dunnett’s post-hoc test was used for multiple comparisons, with beef set as the control group. Differences were significant when *p* < 0.05.

## 3. Results

### 3.1. Moisture and Mineral Composition of Burger Samples

The ingredient compositions of all burgers differed, and these are shown in Table 1. The main vegetable-based constituents of the samples are as follows: A (pumpkin), B (beetroot), C (mycoprotein), D (red cabbage), E (jackfruit), F (potato), G (soy), H (mushroom), and I (beef). The moisture content of raw burgers ranged from 50.5% to 77.9%, whilst cooked burgers ranged between 47.8% and 75.2% (Table 2). Ca, Cu, Fe, Mg, Mn, and Zn content were estimated in the samples. Significant differences in mineral contents were observed between the plant-based burgers versus the beef burger (Table 2). Overall, the plant-based burgers contained higher amounts of Ca, Mg and Mn, whereas beef burgers had lower Cu contents (Table 2).

The ingredient compositions of all burgers varied, and these are shown in Table 1. The first procedure was the determination of the moisture content of each burger, and these ranged between 47.8% and 75.2% (Table 2). Significant differences in mineral content were observed between the vegetarian and vegan burgers versus the beef burger (Table 2). The beef burger had significantly (*p* < 0.001) higher levels of iron than most meat alternatives, except for pumpkin and beetroot, which did not reach significance. The soy burger had significantly higher (*p* < 0.001) Fe than the beef burger (Table 2). Furthermore, the Zn content was significantly (*p* < 0.001) higher in the beef burger compared to all the meat substitutes. All meat alternatives had significantly (*p* < 0.001) higher levels of Ca, Cu, Mg, and Mn than the beef burgers (Table 2).

### 3.2. Iron and Zinc Bioaccessibility from Burger Samples

**Figure 1:**
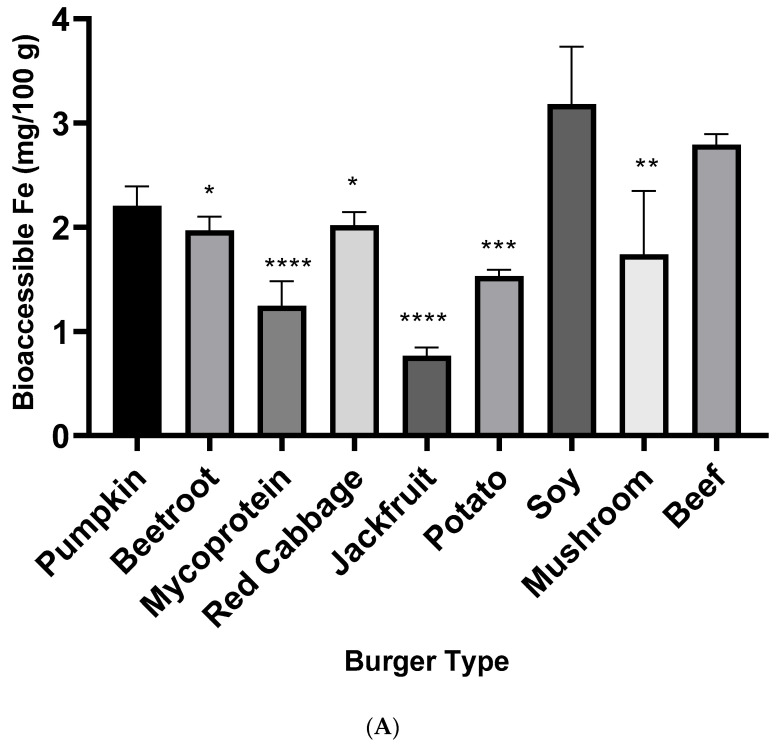

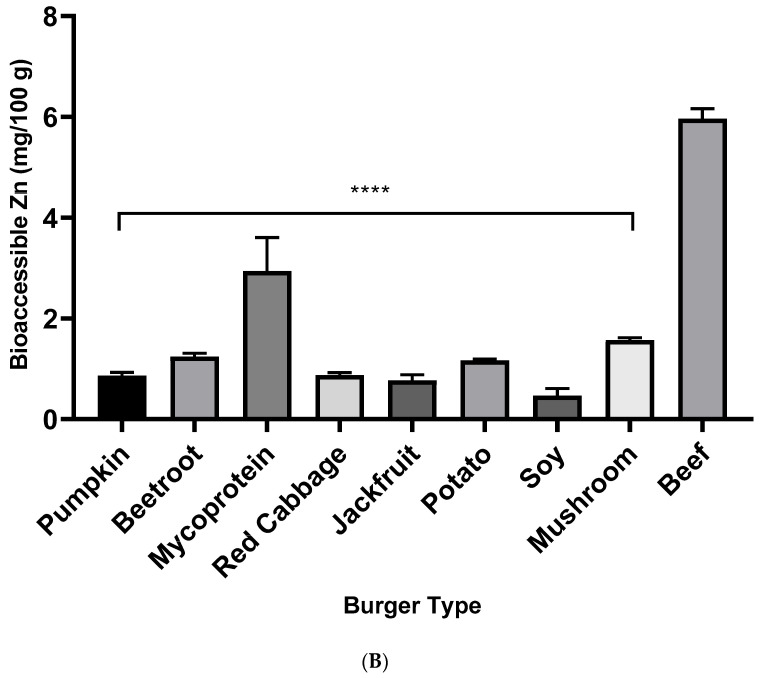
(**A**): Iron bioaccessibility from burgers. A one-way ANOVA and Dunnett’s test were performed to determine significant differences to beef with significance displayed as follows: *p* ≤ 0.05 (*), ≤0.01 (**), ≤0.001 (***), and ≤0.0001 (****). Data represent means (*n* = 3) ± SEM. (**B**): Zinc bioaccessibility from burgers. A one-way ANOVA and Dunnett’s test were performed to determine significant differences to beef with significance displayed as ≤0.0001 (****). Data represent means (*n* = 3) ± SEM.

A well-established in vitro method was employed to estimate the bioaccessibility of minerals from burger samples. Fe bioaccessibility was significantly greater from the beef burger than all other raw burger samples except for soy and pumpkin samples (Figure 1A). Total bioaccessible Zn was significantly (*p* < 0.001) higher from the beef burger than all other burger samples (Figure 1B).

### 3.3. Mineral Uptake from Plant-Based Burger Digests by Caco-2 Cells

**Figure 2:**
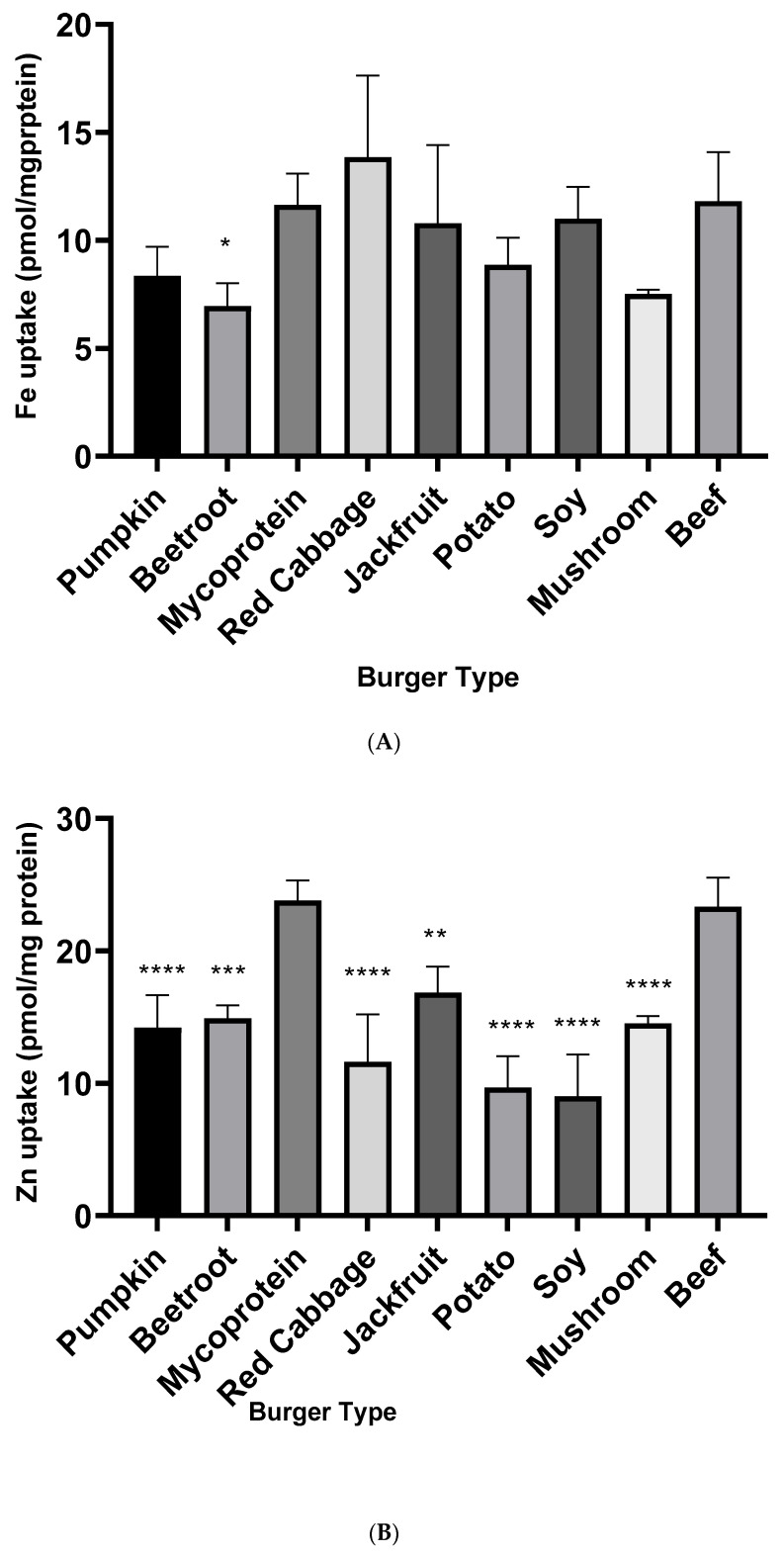
(**A**): Iron bioavailability in Caco-2 cells from burgers. A one-way ANOVA and Dunnett’s test were performed to determine significant differences to beef with significance displayed *p* ≤ 0.05 (*), ≤0.01 (**), ≤0.001 (***), and ≤0.0001 (****). Data represent means (*n* = 4) ± SEM. (**B**): Zinc bioavailability in Caco-2 cells from burgers. A one-way ANOVA and Dunnett’s test were performed to determine significant differences to beef with significance displayed as ≤0.0001 (****). Data represent means (*n* = 4) ± SEM.

Mineral bioavailability is a quantification of the level of minerals taken up by cells after a specified period of exposure. Here, mineral bioavailability was estimated by applying the in vitro digested samples to Caco-2 cells. Fe bioavailability for all plant-based burgers was comparable to the beef burger (I), with the exceptions of the beetroot burgers, which were significantly lower (Figure 2A). The plant-based burgers had significantly less Zn uptake than beef; however, the mycoprotein burger (C) was the only plant-based burger with comparable Zn bioavailability (Figure 2B). 

## 4. Discussion

With meat analogues accounting for approximately 1–2% of the total meat market and increasing by 15% annually in the UK, their consumption, composition, and nutritional content require further investigation [30]. The current study investigated the content and availability of minerals from a selection of meat substitutes sold in the UK. The total mineral content and in vitro availability of Fe and Zn were assessed in the samples. Overall, while iron bioavailability was comparable for most plant-based and beef burgers, zinc availability of the plant-based burgers was lower than for beef.

As expected, significant variability was observed in the mineral content across meat substitutes due to a wide variety of ingredients used in the formulation (Table 1). The mineral content of burger samples was found to be similar to those described in tables from [31]. The soy-based burger contained extremely high levels of iron (14.5 mg Fe/100 g), which is ~4.2-fold higher than that quantified for beef. A recent audit of 50 raw meat-substitute burgers on the Australian market found that they contained an average of 3.6 mg Fe/100 g [32]. Varying Fe levels could be attributed to different cultivation conditions of plant components within the meat substitutes, product development practices, and processing regimens due to wheat flour components of the burgers (which is fortified by law in the UK with iron but not in Australia) [33].

Total zinc content was significantly lower in plant-based burgers (excluding the mycoprotein burger) compared to the beef burger. This result is consistent with the literature, reporting higher total zinc content in ground beef than in plant-based alternatives [34,35]. The data on mycoprotein are also supported by showing that mycoprotein contains comparable amounts of zinc to that found in meat [36,37]. 

Swing et al. [35] quantified mineral levels in various cooked plant-based burgers, comparing them to pork and beef burgers. The results indicated that the plant-based burgers had significantly greater iron and zinc contents than the animal-based burgers. This contrasts with the current study, which showed plant-based burgers contained significantly lower iron and zinc contents compared to the beef burgers. This discrepancy may be related to the differences in the burger components between the two studies. Swing et al. [35] analysed mock meat burgers such as ‘Beyond Meat’, which were made with a variety of ingredients. Interestingly, the report did note that soy-based burgers contained the highest total iron at 36.3 ppm, compared to just 19.4 ppm for beef. 

Our current study found that all meat alternatives have higher calcium levels compared to beef. This could be explained by several factors, such as the potential addition of calcium chloride to plant burgers as a firming agent, the fortification of wheat flour with calcium, and beef being a poor source of calcium (National Diet and Nutrition Survey NDNS, [16]. This suggests that meat substitutes could be an important source of calcium for those who do not consume meat, fish, or dairy products. 

The beef burger had significantly greater iron bioaccessibility than meat substitutes, with the notable exception of soy (Figure 1A). This could be explained in part by the soy burger being fortified with iron. Soy has been reported to lower iron bioaccessibility, due to encapsulation of iron within large peptide aggregates during in vitro digestion [38]. Nonetheless, fortification of soy alongside an adequate quantity of ascorbic acid may partially overcome the inhibitory effect on iron bioaccessibility [39]. In addition, the baking processes carried out on the burgers may also partially reverse soy inhibition of iron [40].

Animal flesh is the primary source of zinc in UK omnivorous diets and represents a source of highly bioavailable zinc [41,42]. Perhaps unsurprisingly, zinc bioaccessibility from the beef burger was significantly greater than from the meat substitutes following in vitro digestion (Figure 1B).

Surprisingly, for most of the plant-based burgers, iron bioavailability did not differ significantly from beef (Figure 2A). This may be related to the additional food components present in the various burgers. For example, soy, which has a naturally high iron content, is an ingredient in several of the plant-based burgers. Furthermore, iron (added as a fortificant) is also listed in the ingredients for some burgers. This suggests that a diet that excludes animal products could achieve adequate intakes of minerals from plant foods; however, content is a relatively poor predictor of bioavailability [43,44]. Thus, total mineral content may not correlate with mineral bioaccessibility and bioavailability.

Only the mycoprotein burger exhibited comparable zinc uptake to beef in Caco-2 cells (Figure 2B). Mycoprotein products have been reported to contain high zinc contents (with a median content of 6.7 mg/100 g) and had very low content of phytate. This suggests that mycoprotein, which is already a dominant meat substitute component for vegans and vegetarians, is equally as effective as beef in supplying adequate zinc amounts in the diets. 

Plant food products are known to contain high levels of phytate, which can reduce iron and zinc uptake from plant-based burgers [45,46]. The inhibitory effect of phytate is potentiated by forming mineral-calcium-phytate complexes of low solubility in the intestinal lumen [17]. Furthermore, the high phytate content in soy products, alongside high dietary calcium intake, has been shown to lower zinc bioavailability in rats [38]. However, in humans, replacing meat by 25% with soy did not impact mineral absorption [47]. This finding might be promising if meat reduction is to be encouraged. However, total replacement of meat with soy led to significantly lower absorption levels of zinc showing that the exclusion of meat may exacerbate poor bioavailability of zinc from plant-derived sources in vegetarian and vegan diets [47]. A recent study estimated the bioavailability of iron and zinc based on the phytate:mineral molar ratio, from samples of meat substitutes bought from convenience stores in Gothenburg, Sweden [48]. None of the products were considered as sources for good mineral bioavailability due to very high content of phytate. The study therefore concluded that the meat substitutes analyzed did not contribute significantly to absorbed iron. The variations in ingredients between the plant-based burgers, as well as the presence and interaction of inhibitors and enhancers in the burgers, can influence mineral bioavailability and provide insight for future investigations. Current dietary guidelines recommend a reduction in red and processed meat consumption and an increase in fruit and vegetable intake to decrease the risk of chronic disease. Therefore, as the population moves more towards the consumption of a plant-based diet, the exploitation of the synergy among dietary components to enhance iron absorption by the incorporation of potent enhancers of mineral absorption in plant burgers becomes increasingly important. 

The composition of the vast array of vegetarian and vegan burgers is constantly changing and developing due to technological advances. Therefore, it is challenging to assess and compare the mineral content and bioavailability of the different products currently available in the UK. Burgers are not eaten in isolation, but often as part of a more complex meal. Consequently, depending on the variety of vegetable constituents of plant-based burgers, they could provide adequate quantities of iron and zinc in the diet. 

A limitation of the current work is that cultured cell models may not reflect true physiological responses. However, Caco-2 cells have been validated and verified for the determination of in vitro iron bioavailability because the cells express intestinal microvilli enzymes and differentiation markers that mimic human small intestine enterocytes [27,49]. Moreover, the lack of modulators of systemic factors of iron absorption could confound the extrapolation to in vivo iron absorption studies [50]. Nevertheless, assessments of the bioaccessibility of iron and its bioavailability in Caco-2 cells are useful for predicting the relative bioavailability of a wide range of plant foods. This information can be used to select specific food products for further evaluation in human dietary interventions to measure the bioavailability of minerals from meals containing meat substitutes.

## 5. Conclusions

In summary, except for soy and mycoprotein burgers, the total iron and zinc contents of the plant-based burgers were significantly lower than those from the beef. Likewise, zinc bioavailability was significantly lower from plant-based burgers, while iron bioavailability was comparable between the meat-containing and the meat-substituted burgers. Thus, individuals consuming plant-based diets need to be aware not only of the foods that are rich in iron and zinc, but also of the foods to eat in conjunction to maximise mineral bioavailability. Only the mycoprotein-based burger showed comparable zinc bioavailability to beef, and thus it could be suitable alternative to a traditional meat-containing burger. Future studies should now investigate the nutritional and health effects of meat substitutes. 

## Figures and Tables

**Table 1:** Ingredient composition of burger samples.

Sample	Ingredient List
**A**	Pumpkin (50%), sweet potato, soya protein, sunflower oil, potato starch, methyl cellulose, coriander, turmeric, white pepper, cumin, fenugreek, wheat fibre, salt, paprika powder, onion powder, garlic powder, red paprika
**B**	Beetroot (80%), potato starch, onion, soya protein, wheat fibres, methyl cellulose, salt, potato protein, garlic purée
**C**	Mycoprotein^TM^ (38%), textured wheat protein (wheat flour, sodium alginate, plain caramel), rehydrated free range egg white, vegetable oil, onion, smoked yeast, potassium chloride, milk proteins, roasted barley, calcium chloride, calcium acetate
**D**	Red cabbage, black turtle beans, breadcrumbs (wheat flour, calcium carbonate, iron, niacin, thiamin), sunflower oil, salt, yeast, rosemary, soya protein concentrate, sushi rice, carrot, rice vinegar, water, onion, dark soya sauce, beetroot, rapeseed oil, red chilli purée, red miso, maple syrup, garlic, black pepper
**E**	Jackfruit (45%), water, rice flour, gram flour, maize flour, maize starch, yeast extract, brown sugar, tomato paste, cornflour, potato starch, salt, sunflower oil, red pepper, spirit vinegar, muscovado sugar, cider vinegar, parsley, onion powder, garlic powder, lemon powder, sodium bicarbonate, disodium diphosphate, paprika, cane molasses, grape vinegar, fennel seed, cayenne pepper, cumin, coriander, rosemary, dextrose monohydrate, pepper, xanthan gum, black treacle, tamarind paste, pimento, ginger, cloves
**F**	Potato (15%), aubergine, onion, lentils, rapeseed oil, feta, spring onion, chickpeas, sweet potato, egg, cornflour, potato starch, wheat flour, calcium carbonate, iron, niacin, thiamin, garlic purée, red chilli purée, parsley, mint, salt, cumin, extra virgin olive oil, tomato purée, pepper, coriander, paprika, yeast
**G**	SOY structure (Water, soya protein, wheat starch, wheat protein) (64%), broad beans, sunflower oil, palm oil, onion powder, pepper, garlic, ginger, mace, onion, clove, coriander, oregano, methyl cellulose, bamboo fibre, tapioca starch, seaweed, salt, iron, vitamin B12
**H**	Water, mushrooms (32%), wheat gluten, pea flour, wheat flour, vegetable suet, pea fibre, methylcellulose, pea starch, salt, onion, yeast, pepper, sodium metabisulphite
**I**	Beef (95%), onion, salt, butter, pepper, sodium sulphite, sodium ascorbate

Vegetarian: C, F, & G; Vegan: A, B, D, E, & H; Animal: I. Ingredient list was reproduced from labels on the burger samples purchased from a supermarket in the UK.

**Table 2:** Mean moisture and mineral contents of vegetarian, vegan, and beef burgers (mg/100 g) dry weight.

Sample	Moisture (%)	Fe	Ca	Cu	Mg	Mn	Zn
**A**	67.9	3.36 ± 0.03	134 ± 0.93 ***	0.43 ± 0.01 ***	85.9 ± 0.34 ***	1.20 ± 0.12 ***	1.10 ± 0.02 ***
**B**	75.2	3.39 ± 0.06	156 ± 1.38 ***	0.42 ± 0.01 ***	94.1 ± 0.70 ***	1.88 ± 0.01 ***	1.67 ± 0.03 ***
**C**	62.2	1.13 ± 0.04 ***	260 ± 7.01 ***	0.56 ± 0.01 ***	50.5 ± 0.54 ***	3.25 ± 0.04 ***	7.15 ± 0.08 *
**D**	54.3	2.70 ± 0.05 ***	93.4 ± 1.39 ***	0.33 ± 0.00 ***	70.4 ± 0.96 ***	0.97 ± 0.01 ***	1.14 ± 0.04 ***
**E**	57.1	1.27 ± 0.03 ***	31.7 ± 0.10 ***	0.24 ± 0.00 ***	40.1 ± 0.20 ***	0.63 ± 0.00 ***	0.89 ± 0.02 ***
**F**	47.8	1.99 ± 0.08 ***	57.9 ± 0.92 ***	0.23 ± 0.00 ***	39.5 ± 0.34 ***	0.47 ± 0.01 ***	0.91 ± 0.03 ***
**G**	53.6	14.5 ± 0.03 ***	150 ± 1.32 ***	0.28 ± 0.00 ***	62.5 ± 0.46 ***	0.87 ± 0.01 ***	1.14 ± 0.01 ***
**H**	61.2	2.90 ± 0.02 ***	46.2 ± 0.28 ***	0.43 ± 0.00 ***	44.5 ± 0.15 ***	0.83 ± 0.01 ***	2.24 ± 0.01 ***
**I**	54.1	3.42 ± 0.01	9.59 ± 0.21	0.14 ± 0.00	37.2 ± 0.12	0.07 ± 0.00	7.41 ± 0.06

Mineral content quantified by ICP-OES. The main ingredients represented by the sample coding are as follows: A: Pumpkin, B: Beetroot, C: Mycoprotein, D: Red cabbage, E: Jackfruit, F: Potato, G: Soy, H: Mushroom, I: Beef. Data expressed as mean ± SEM (*n* = 5). A one-way ANOVA and Dunnett’s test were performed to determine significant differences to beef. * Significant at the 0.05 probability level; *** Significant at the 0.001 probability level.

## Data Availability

The data are not publicly available due to privacy reason.
